# *Lnc-THOR* silencing inhibits human glioma cell survival by activating MAGEA6-AMPK signaling

**DOI:** 10.1038/s41419-019-2093-0

**Published:** 2019-11-14

**Authors:** Jun Xue, Shan Zhong, Bo-min Sun, Qing-Fang Sun, Liang-Yun Hu, Si-Jian Pan

**Affiliations:** 1 Department of Neurosurgery, Rui-Jin Hospital, Shanghai Jiao-Tong University School of Medicine, 200025 Shanghai, P. R. China; 2 Department of Stereotactic and Functional Neurosurgery, Rui-Jin Hospital, Shanghai Jiao-Tong University School of Medicine, 200025 Shanghai, P. R. China

**Keywords:** RNA, Targeted therapies

## Abstract

*Long non-coding RNA THOR* (*Lnc-THOR*) binds to IGF2BP1, essential for its function. We here show that *Lnc-THOR* is expressed in human glioma tissues and cells. Its expression is extremely low or even undetected in normal brain tissues, as well as in human neuronal cells and astrocytes. We show that Lnc-THOR directly binds to IGF2BP1 in established and primary human glioma cells. shRNA-mediated *Lnc-THOR* knockdown or CRISPR/Cas9-induced *Lnc-THOR* knockout potently inhibited cell survival and proliferation, while provoking glioma cell apoptosis. Contrarily, forced overexpression of *Lnc-THOR* promoted glioma cell growth and migration. Importantly, *Lnc-THOR* shRNA or knockout activated MAGEA6-AMPK signaling in glioma cells. AMPK inactivation, by AMPKα1 shRNA, knockout, or dominant-negative mutation (T172A), attenuated *Lnc-THOR* shRNA-induced A172 glioma cell apoptosis. Moreover, CRISPR/Cas9-induced IGF2BP1 knockout activated MAGEA6-AMPK signaling as well, causing A172 glioma cell apoptosis. Significantly, *Lnc-THOR* shRNA was ineffective in IGF2BP1 KO A172 cells. In vivo, *Lnc-THOR* silencing or knockout potently inhibited subcutaneous A172 xenograft tumor growth in mice. MAGEA6 downregulation and AMPK activation were detected in *Lnc-THOR*-silenced/-KO A172 tumor tissues. Taken together, *Lnc-THOR* depletion inhibits human glioma cell survival possibly by activating MAGEA6-AMPK signaling.

## Introduction

Glioma is among the most aggressive human malignancies, causing significant human mortalities each year^1–3^. In the clinical practices, gliomas are commonly diagnosed at late/advanced stages with extremely poor prognosis^3^. Molecularly targeted therapies are essential for better glioma prognosis^4–6^. Our group has been exploring novel therapeutic targets for this devastating disease^7–10^. *Non-coding RNAs* (*ncRNAs*), including *microRNAs*, *long non-coding RNAs* (*LncRNA*), and *circular RNAs*, are originally known as transcriptional noise. Recent studies have implied that *LncRNAs*, and other *ncRNAs*, play pivotal roles in initiation and progression of human glioma^11^ and many other cancers^12–14^.

A recent study by Hosono et al. has reported a conserved *LncRNA*, *THOR* (“*Lnc-THOR*”)^15^. Its expression is detected in testis, and also in a number of different human cancers^15–20^. *Lnc-THOR* knockdown or knockout (KO) potently inhibited human cancer cell survival^15^. *Lnc-THOR* directly associates with insulin-like growth factor 2 (IGF2) mRNA-binding protein 1 (IGF2BP1), a conserved RNA-binding family protein^15^. *Lnc-THOR* association is essential for IGF2BP1’s function, as well as stabilization of IGF2BP1 target *mRNAs*, including *IGF2*, *Gli1* (*glioma-associated oncogene homolog 1*), *Myc*, and *CD44*^15,20^.

Our previous studies have implied that forced activation of AMP-activated protein kinase (AMPK) can inhibit human glioma cells^21,22^. Thr-172 phosphorylation of AMPKα1 is essential for AMPK activation. AMPK activation inhibits mammalian target of rapamycin (mTOR) complex 1 (mTORC1), a key oncogenic cascade^23,24^. In human cancer cells, activated AMPK could also induce growth inhibition and cell-cycle arrest by stabilizing and activating p53^25^. Moreover, AMPK activation is shown to trigger autophagy and degradation of multiple growth factor receptors (i.e., epidermal growth factor receptor (EGFR) and platelet-derived growth factor receptor α (PDGFRα)), thereby causing cancer cell inhibition^26^.

AMPKα1 expression is often sequestered in human cancer cells. Pineda et al. showed that MAGEA6-TRIM28 complex is a cancer-specific ubiquitin ligase, responsible for degradation AMPKα1 only in cancer cells^27^. We have previously shown that MAGEA6 knockdown by targeted short hairpin RNA (shRNA) restored AMPKα1 expression, causing glioma cell death and apoptosis^21^. In the present study, we will show that *Lnc-THOR*-IGF2BP1 cascade is essential for MAGEA6 expression in glioma cells. Inhibition of *Lnc-THOR*-IGF2BP1 cascade will induce MAGEA6 downregulation, AMPKα1 expression, and AMPK signaling activation, inhibiting glioma cell survival in vitro and in vivo.

## Materials and methods

### Chemicals and reagents

Puromycin and neomycin were obtained from Sigma-Aldrich (St. Louis, MO). Cell culture reagents were provided by Gibco-BRL (Grand Island, NY). The anti-MAGEA6 antibody (ab38495) was purchased from Abcam (Shanghai, China). All other antibodies were provided by Cell Signaling Tech (Shanghai, China). TRIzol reagents for RNA assays, Lipofectamine 2000, and other transfection reagents were obtained from Invitrogen (Shanghai, China).

### Cell culture

Cultures of HCN-1a human neuronal cells, A172 and U251MG (“U251”) human glioma cells, as well as the primary human astrocytes, were described earlier^22,28^. The human glioma cells, derived from two primary glioma patients, were provided by Dr. Cao^29,30^, which were named as “Pri-1/Pri-2,” and cultured as previously described^30,31^. The protocols of studying human cells and tissues were approved by the Ethics Review Board of Shanghai Jiao-Tong University School of Medicine, according to Declaration of Helsinki.

### Human tissues

As reported earlier^21^, a total of five glioma tissues, along with paired surrounding normal brain tissues, were acquired and stored in liquid nitrogen. Tissues were separated, thoroughly washed, minced, and homogenized by the tissue lysis buffer (BiYunTian, Wuxi, China). Written informed consent was obtained from each participant.

### Quantitative real-time reverse transcriptase polymerase chain reaction (qPCR)

As reported^21^, 500 ng RNA of each sample was applied in the reverse transcription (RT) reaction with specific RT primers and superscript III reverse transcriptase (Invitrogen). Afterwards, 100 ng obtained complementary DNA (cDNA) template was mixed with SYBR Master Mix (Applied Biosystem) and 200 nM primers. We utilized ABI Prism 7600H Fast Real-Time PCR system for qPCR assays. The primers are listed in Table 1. qPCR quantification was through 2^−ΔCt^ method using the following formula: 2^−(Ct of target gene − Ct of reference gene)^. qPCR primers are listed in Table 1.

**Table 1:** Primers utilized in this study

Gene name	Forward primers	Reverse primers
*GAPDH*	5’-CACCACCATGGAGAAGGCTGG-3’	5’-GAAGTCAGAGGAGACCACCTG-3’
*MAGEA6*	5’-TGGAGGACCAGAGGCCCCC-3’	5’-CAGGATGATTATCAGGAAGCCTGT-3’
*Lnc-THOR*	5’-*CAAGGTGCTTCTCTCTGGATTT*-3’	5’-*GCCAAAGTCATTTGTTGGGTAT*-3’
*U6*	5′-CTCGCTTCGGCAGCACATATACT-3′	5′-ACGCTTCACGAATTTGCGTGTC-3′
*AMPKα1*	5′-AGGAAGAATCCTGTGACAAGCAC-3′	5′-CCGATCTCTGTGGAGTAGCAGT-3′
*Gli1*	5’-AGCCTTCAGCAATGCCAGTGAC-3’	5’-GTCAGGACCATGCACTGTCTTG-3’
*Myc*	5’-CCTGGTGCTCCATGAGGAGAC-3’	5’-CAGACTCTGACCTTTTGCCAGG-3’
*IGF2*	5’-TGGCATCGTTGAGGAGTGCTGT-3’	5’-ACGGGGTATCTGGGGAAGTTGT-3

### Lnc-THOR shRNA

A set of two shRNAs, against non-overlapping sequence of *Lnc-THOR* (“Seq1/2,” designed and verified by Genechem, Shanghai, China), were individually inserted into GV248 construct. The construct, along with the lentivirus package plasmids (Genechem), were transfected to HEK-293 cells to generate *Lnc-THOR* shRNA lentivirus. The virus was enriched, filtered, and added to glioma cells (plated at a density of 1 × 10^5^ cells/well into 6-well plates). Cells were then subjected to selection by using puromycin (2.5 μg/mL, for 10–12 days). In stable cells, *Lnc-THOR* knockdown was verified by qPCR assay.

### *Lnc-THOR* KO

The CRISPR/Cas9 *Lnc-THOR* KO construct (with *sgRNA*, 5’-*CACCgAGGGTGTAGCGCGGGCTAGA*-3’, R: 5’-*AAACTCTAGCCCGCGCTACACCCTc*-3^15^) was provided by Dr. Liang, which was transfected to glioma cells (plated at a density of 1 × 10 ^5^ cells/well into 6-well plates) by Lipofectamine 2000 reagents. Fluorescent-activated cell sorting (FACS)-mediated sorting of the green fluorescent protein (GFP)-positive cells were performed to select monoclonal cells, which were then cultured in the puromycin-containing complete medium to achieve stable cells. *Lnc-THOR* KO was verified by qPCR assay.

### *Lnc-THOR* overexpression

The full-length *Lnc-THOR* was amplified by the described primers^15^ and inserted to the GV248 lentiviral construct (Genechem). The lentiviral GV248-*Lnc-THOR* construct (“LV-*Lnc-THOR*”) was transfected to glioma cells (plated at a density of 1 × 10^5^ cells/well into 6-well plates), followed by selection using puromycin (2.5 μg/mL) for 10–12 days. In stable cells, *Lnc-THOR* overexpression was verified by qPCR assay.

### Cell viability assay

Briefly, cells were plated at a density of 3 × 10^3^ cells/well into 96-well plates. Following culture of 96 h, 3-[4,5-dimethylthiazol-2-yl]-2,5 diphenyl tetrazolium bromide (MTT; 5 mg/mL, 20 μL/well, dissolved in phosphate-buffered saline (PBS)) was added, cells were further incubated for additional 2 h, and its optical density (OD) was tested at 590 nm.

### Cell proliferation assays

For the soft agar colony-formation assay, A172 cells (5000 cells of each treatment) were re-suspended in agar (0.5%)-containing complete medium (with fetal bovine serum (FBS)) and added on the top of 10-cm culture dishes. After incubation for 10 days, A172 cell colonies were stained and manually counted. The detailed protocol for the 5-ethynyl-2’-deoxyuridine (EdU) staining assay was reported earlier^32^.

### Apoptosis assays

The detailed protocols of apoptosis assays, including Histone DNA enzyme-linked immunosorbent assay and Annexin V FACS, as well as terminal deoxynucleotidyl transferase-mediated dUTP-fluorescein nick end labeling (TUNEL) staining assay and caspase-3/caspase-9 activity assays, were described in previous studies^33,34^.

### “Transwell” in vitro migration assay

A172 glioma cells (3 × 10^5^ cells in 300 μL medium) were seeded into the upper part of the “Transwell” chambers (12-μm pore size, BD Biosciences). The lower compartments were filled with complete medium with 10% FBS. After 48 h, on the upper surfaces the non-migrated A172 cells were removed. On the lower surfaces, the migrated cells were fixed, stained, and counted.

### Western blotting analysis

The detail protocol of western blotting assay was described in our previous studies^9,10^. Briefly, for each treatment 40 μg of protein lysates (in each lane) were separated in denaturing 10–12% polyacrylamide gels and transferred to a polyvinylidene difluoride blots. After blocking (in 10% milk PBST solution) and three washes in TBST, blots were incubated with the indicated primary and secondary antibodies. Immuno-reactive proteins were detected by an enhanced chemiluminescence kit (Amersham, Shanghai, China) and analyzed through autoradiography. ImageJ software (NIH) was utilized for the quantification of the protein band, which was always normalized to the loading control.

### AMPKα1 shRNA

As described^21^, the lentiviral AMPKα1 shRNA was added to A172 cells (plated at a density of 1 × 10^5^ cells/well into 6-well plates) for 48 h. Puromycin (2.5 μg/mL)-containing complete medium was added to select stable cells for 5–6 days. Control cells were infected with the lentiviral scramble control shRNA (“sh-C”). AMPKα1 silencing in the stable cells was confirmed by western blotting.

### AMPKα1 dominant-negative mutation

The dominant-negative AMPKα1 (dnAMPKα1, T172A, as reported^21^) or the empty vector (pSuper-neo-Flag) was transfected to A172 cells (plated at a density of 1 × 10^5^ cells/well into 6-well plates) by Lipofectamine 2000. Neomycin (1.0 μg/mL) was added to select stable cells for 5–6 days. Expression of the mutant AMPKα1 was verified by western blotting.

### AMPK activity assay

Following the treatments, 200 μg of total cellular lysates were first incubated with anti-AMPKα1 antibody. The AMPK activity was examined in the kinase assay buffer by adding AMP-[γ-^32^P] ATP mixture and AMPK substrate SAMS (HMRSAMSGLHLVKRR) peptide^35^. Phosphocellulose paper was added afterwards, stopping the reactions. The AMPK radioactivity was examined by a scintillation counter, and its value was normalized to control level.

### IGF2BP1 or AMPKα1 KO

A172 cells were seeded onto 6-well tissue culture plates at a density of 1 × 10 ^5^ cells/well. The lenti-CRISPR/Cas9-IGF2BP1-KO-GFP construct (provided by Dr. Zhao^36^) or the lenti-CRISPR/Cas9-AMPKα1-KO-GFP construct (from Dr. Li^37^) was transfected to A172 cells through Lipofectamine 2000 protocol. FACS-mediated sorting of the GFP-positive cells were performed to select the monoclonal cells, which were then cultured in the puromycin-containing complete medium to achieve stable cells. IGF2BP1 or AMPKα1 KO in the stable cells was confirmed by western blotting and/or qPCR assays.

### Ectopic IGF2BP1 overexpression

The recombinant adenovirus encoding IGF2BP1 expression construct (provided by Dr. Zhao^36^) was added to cultured A172 cells (plated at a density of 1 × 10^5^ cells/well into 6-well plates) for 48 h. Cells were thereafter subjected to puromycin (2.5 μg/mL) selection for another 5–6 days. IGF2BP1 overexpression was confirmed by western blotting.

### RNA immunoprecipitation (RIP)

RIP experiments were carried out through a described protocol^38^. Briefly, glioma cells were trypsinized, washed, and incubated with 0.3% formaldehyde and glycine^39^. Afterwards, glioma cells were washed, and resuspended, with the pellets dissolved in the RIP buffer^38^. The lysates were then incubated with the anti-IGF2BP1 antibody. Pellets were washed, re-suspended, and incubated with proteinase K-containing buffer. IGF2BP1-bound *Lnc-THOR* and *MAGEA6 mRNA* was tested by qPCR, with its level normalized to internal controls.

### RNA Pull-Down assay

RNA Pull-Down was carried out using a previously described protocol^39^. In short, the biotin-labeled full-length *Lnc-THOR* (provided by Dr. Wang^39^) was folded in RNA structure buffer and incubated with cleared nuclei lysates of the glioma cells together with Dynabeads MyOne Streptavidin C1 magnetic beads (“Beads,” again provided by Dr. Wang^39^). Beads were washed, with the retrieved proteins examined by western blotting.

### Xenograft assay

As reported^21^, the female severe combined immunodeficient (SCID) mice were housed under standard procedures. *Lnc-THOR* shRNA-bearing stable A172 cells, *Lnc-THOR* KO stable A172 cells, or the parental control A172 cells (5 × 10^6^ cells in 200 µl of Matrigel gel, no serum, each mouse) were subcutaneously (s.c.) injected to the flanks. When the volume reached approximately 100 mm^3^ for each tumor (“Day-0”), the recordings were started. Tumor volumes were calculated as described^21^. All animal procedures were approved by IACUC of Shanghai Jiao-Tong University School of Medicine.

### Statistical analysis

All statistics were calculated by using the SPSS 18.0 statistical software (SPSS, Chicago, IL). Descriptive statistics including mean and standard deviation (SD) along with one-way analyses of variance were applied to determine significant differences. Two-tailed unpaired *T* test (Excel 2013) was applied to test significance between the two treatment groups. *p* < 0.05 was considered significant.

## Results

### *Lnc-THOR* expression in human glioma tissues and cells

**Fig. 1:**
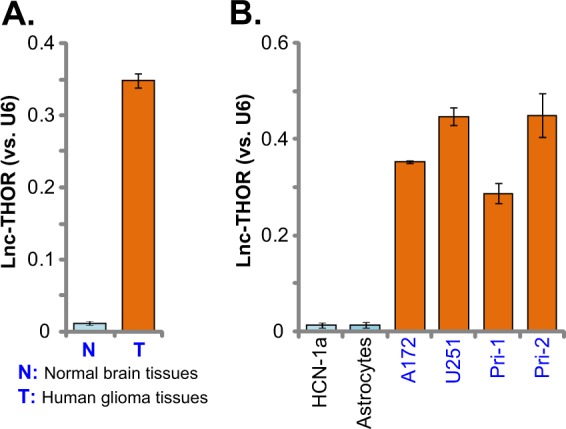
*Lnc-THOR* expression in human glioma tissues and cells. *Lnc-THOR* expression levels in human glioma tissues (“T,” *n* = 5) and paired surrounding normal brain tissues (“N,” *n* = 5) were tested by quantitative real-time PCR (qPCR) (**a**). The primary human astrocytes (“Astrocytes”), HCN-1a neuronal cells, as well as A172 and U251MG (“U251”) glioma cell lines and primary human glioma cells (“Pri-1/Pri-2”), were subjected to qPCR assay of *Lnc-THOR* expression (**b**). Data are presented as mean ± SD (*n* = 5). Experiments in this figure were repeated three times, and similar results were obtained

First, we tested the expression of *Lnc-THOR* in human glioma tissues. As described in our previous studies^21^, a total of five pairs of human glioma tissues (“T”) and paired surrounding normal brain tissues (“N”) were analyzed, and qPCR assay results in Fig. 1a show that *Lnc-THOR* levels are high in human glioma tissues, whereas its levels in normal brain tissues are, however, extremely low (Fig. 1a). Further studies show that *Lnc-THOR* is expressed in established (A172 and U251 lines) and primary human glioma cells (derived from two different patients, “Pri-1/-2”) (Fig. 1b). Its expression is almost undetected in the primary human astrocytes^22^ and HCN-1a neuronal cells^22^ (Fig. 1b). These results confirm unique *Lnc-THOR* expression in human glioma tissues and cells.

### *Lnc-THOR* silencing or KO inhibits human glioma cell progression in vitro

**Fig. 2:**
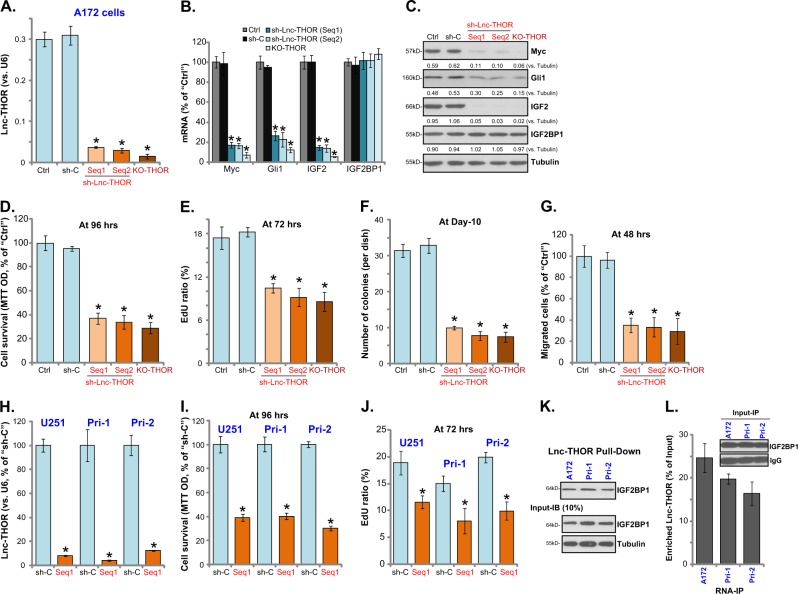
*Lnc-THOR* silencing or KO inhibits human glioma cell survival and proliferation. The genetically modified stable A172 cells, with *Lnc-THOR* shRNA (“sh-Lnc-THOR,” with non-overlapping sequences, “Seq1/2”), scramble non-sense control shRNA (“sh-C”), or the lenti-CRISPR/Cas9 Lnc-THOR-KO construct (“KO-THOR”), were established, the expression of *Lnc-THOR* and listed genes in the stable cells and in parental control A172 cells (“Ctrl”) were tested by qPCR and western blotting assays (**a**–**c**); Cells were further cultured for the indicated time, and cell viability was tested by MTT assay (**d**); Cell proliferation was analyzed by EdU staining (**e**) and soft agar colony-formation (**f**) assays; Cell migration was tested by “Transwell” assays (**g**). U251MG (“U251”) and primary human glioma cells (“Pri-1/Pri-2”) were transfected with lentiviral *Lnc-THOR* shRNA (“sh-Lnc-THOR,” “Seq1”) or “sh-C” and stable cells were established via puromycin selection. *Lnc-THOR* levels were tested by qPCR assay (**h**); Cell survival and proliferation were tested by MTT assay (**i**) and EdU staining assay (**j**), respectively. Western blotting assay of the IGF2BP1 protein retrieved by in vitro-transcribed *Lnc-THOR* in A172 cells and primary human glioma cells (**k**). qPCR analyses of *Lnc-THOR* expression enriched by the IGF2BP1 protein in A172 cells and primary human glioma cells (**l**). For all the in vitro function assays, the exact same amount of viable cells was initially seeded in each well/dish (“0 h”) (same for all figures). Blot data were quantified and normalized to the corresponding loading control (**c**). Data are presented as mean ± SD (*n* = 5). *p < 0.05 vs. “sh-C” cells. Experiments in this figure were repeated three times, and similar results were obtained

In order to study the function of *Lnc-THOR* in human glioma cells, two lentivirus-encoded *Lnc-THOR* shRNAs, with non-overlapping sequences (“Seq1/2”), were individually transfected to A172 glioma cells. Following puromycin selection, the stable cells were established (“sh-Lnc-THOR” cells). Moreover, the lenti-CRISPR/Cas9 *Lnc-THOR*-KO construct (see “Methods” section) was transfected to A172 cells. Stable cells (“KO-THOR” cells) were established by FACS sorting of GFP cells and puromycin selection. Analyzing *Lnc-THOR* expression in the stable cells, by qPCR, confirmed that *Lnc-THOR* levels were dramatically downregulated in the stable cells with *Lnc-THOR* shRNA or *Lnc-THOR* KO construct (Fig. 2a). *Lnc-THOR* binds to IGF2BP1 to ensure mRNA stabilization of key pro-cancerous genes, including *IGF2*, *Gli1*, and *Myc*^15,16,18,20^. In A172 glioma cells, mRNA levels of *IGF2*, *Gli1*, and *Myc* were significantly downregulated in *Lnc-THOR*-silenced or *Lnc-THOR*-KO A172 cells (Fig. 2b). IGF2, Gli1, and Myc proteins were downregulated as well (Fig. 2c). *Lnc-THOR* shRNA or KO did not affect *IGF2BP1 mRNA* (Fig. 2b) and protein expression (Fig. 2c). The scramble non-sense control shRNA (“sh-C”) had no significant effect on the expression of *Lnc-THOR*-IGF2BP1 pathway genes (Fig. 2a–c).

MTT, EdU staining, and soft agar colony-formation assays were performed to test glioma cell functions. When compared to control A172 cells, in *Lnc-THOR*-silenced or *Lnc-THOR*-KO A172 cells, MTT OD values (Fig. 2d), EdU percentages (Fig. 2e), and the number of colonies (Fig. 2f) were significantly decreased. A172 cell in vitro migration, tested by the “Transwell” assays, were significantly inhibited by *Lnc-THOR* shRNA or KO (Fig. 2g). In U251MG cells and primary human glioma cells (“Pri-1/-2”), transfection of the lentiviral *Lnc-THOR* shRNA (“Seq1”) induced >90% reduction of *Lnc-THOR* expression (Fig. 2h), causing reduced MTT OD (Fig. 2i) and EdU ratio (Fig. 2j). These results show that *Lnc-THOR* silencing or KO inhibits glioma cell growth and migration.

*Lnc-THOR*-IGF2BP1 binding has been reported in other cancer cells^15,20,39^. To test the direct association between *Lnc-THOR* and the IGF2BP1 protein in glioma cells, we employed a *Lnc-THOR* pull-down assay^39^. Results demonstrated that the IGF2BP1 protein is co-precipitated with the in vitro-transcribed biotinylated *Lnc-THOR* (provided by Dr. Wang^39^) in both A172 cells and primary human glioma cells (“Pri-1/-2”) (Fig. 2k). In addition, the RIP assay results show again the direct binding between *Lnc-THOR* and the IGF2BP1 protein in A172 cells and the primary human glioma cells (Fig. 2l).

### *Lnc-THOR* silencing or KO induces apoptosis activation in human glioma cells

**Fig. 3:**
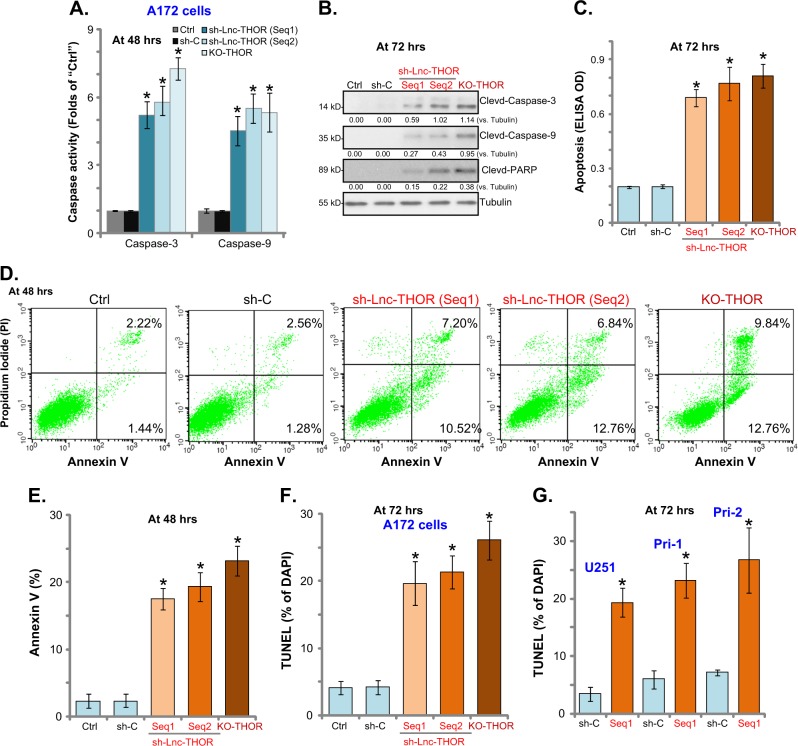
*Lnc-THOR* silencing or KO induces apoptosis activation in human glioma cells. The genetically modified stable A172 cells, with *Lnc-THOR* shRNA (“sh-Lnc-THOR,” with non-overlapping sequences, “Seq1/2”), scramble non-sense control shRNA (“sh-C”), or the lenti-CRISPR/Cas9 Lnc-THOR-KO construct (“KO-THOR”), were established; caspase-3/-9 activity and apoptosis activation in the stable cells and in parental control A172 cells (“Ctrl”) were tested by the listed assays (**a**–**f**). U251MG (“U251”) and primary human glioma cells (“Pri-1/Pri-2”) were transfected with *Lnc-THOR* shRNA (“sh-Lnc-THOR,” “Seq1”) or “sh-C,” stable cells were established via puromycin selection, and cell apoptosis was quantified by the TUNEL staining (**g**). Blot data were quantified and normalized to the corresponding loading control (**b**). Data were presented as mean ± SD (*n* = 5). *p < 0.05 vs. “sh-C” cells. Experiments in this figure were repeated three times, and similar results were obtained

The potential effect of *Lnc-THOR* on glioma cell apoptosis was studied. As shown, in the A172 cells with *Lnc-THOR* shRNA (“Seq-1/-2”) or Lnc-THOR-KO construct (“KO-THOR” cells, see Fig. 2), the activities of caspase-3 and caspase-9 were significantly increased (compared to control A172 cells, Fig. 3a). Furthermore, *Lnc-THOR* silencing or KO in A172 cells induced cleavages of caspase-3, caspase-9, and PARP (poly ADP-ribose polymerase) (Fig. 3b), as well as accumulation of histone-bound DNA (Fig. 3c). In addition, Annexin V percentages (Fig. 3d, e) and nuclear TUNEL ratios (Fig. 3f) were significantly increased in *Lnc-THOR*-silenced or -KO cells. In U251MG and primary human glioma cells (“Pri-1/-2”), the lentiviral *Lnc-THOR* shRNA (“Seq1”) similarly induced increases of nuclear TUNEL ratios (Fig. 3g). Taken together, these results clearly show that *Lnc-THOR* silencing or KO provokes apoptosis activation in human glioma cells.

### *Lnc-THOR* overexpression promotes human glioma cell survival and proliferation

**Fig. 4:**
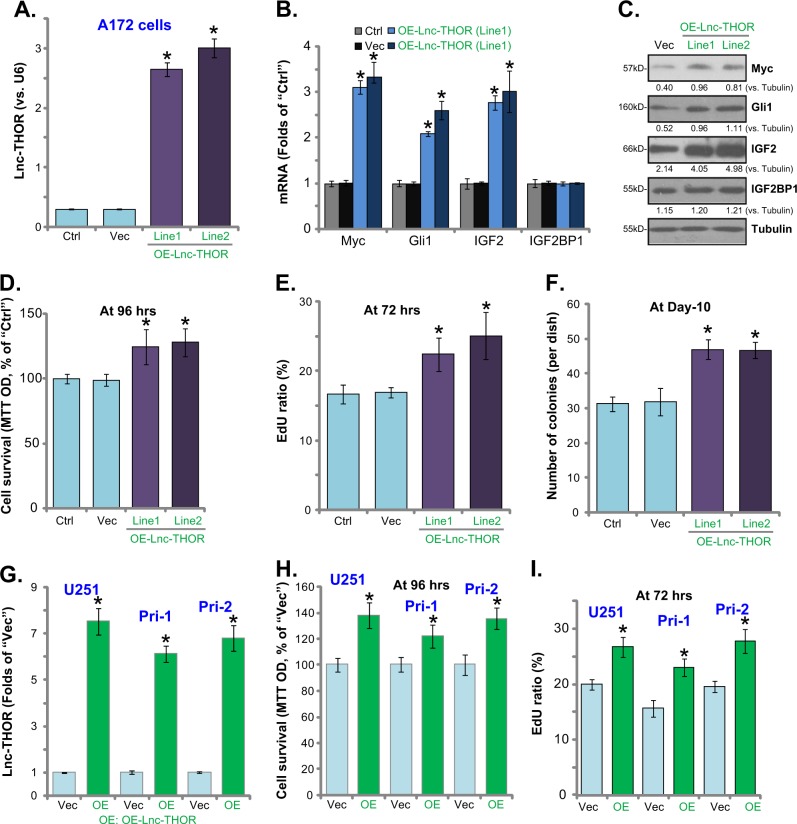
*Lnc-THOR* overexpression promotes human glioma cell survival and proliferation. The genetically modified stable A172 cells, with lentiviral *Lnc-THOR* expression construct (“OE-Lnc-THOR,” two lines, “Line1/2”) or empty vector (“Vec”), were established, and the expression of *Lnc-THOR* and listed genes in the stable cells and in parental control A172 cells (“Ctrl”) were tested by qPCR and western blotting assay (**a**–**c**); Cells were further cultured for the indicated time, and cell viability was tested by MTT assay (**d**); Cell proliferation was analyzed by EdU staining (**e**) and soft agar colony-formation assay (**f**). U251MG (“U251”) and primary human glioma cells (“Pri-1/Pri-2”) were infected with lentiviral Lnc-THOR expression construct (“OE”) or empty vector (“Vec”), and *Lnc-THOR* expression (**g**), cell viability (**h**), and proliferation (**i**) were tested. Blot data were quantified and normalized to the corresponding loading control (**c**). Data were presented as mean ± SD (*n* = 5). *p < 0.05 vs. “Vec” cells. Experiments in this figure were repeated three times, and similar results were obtained

Since *Lnc-THOR* silencing or KO inhibited glioma cell growth and migration, we hypothesized that forced overexpression of *Lnc-THOR* shall exert opposite functions. To test this hypothesis, the lentivirus encoding *Lnc-THOR* expression construct (see “Methods”) was transduced to A172 glioma cells. Following selection using the puromycin-containing medium, two A172 cell lines were established (“Line1/2”). Testing *Lnc-THOR* expression, by qPCR, confirmed that *Lnc-THOR* levels were significantly increased in the stable cells with *Lnc-THOR* construct (“OE-Lnc-THOR” cells). Consequently, mRNA and protein expression of IGF2BP1 targets, *IGF2*, *Gli1*, and *Myc*, were upregulated (Fig. 4b, c). IGF2BP1 expression was again not changed (Fig. 4b, c). As compared to vector control cells, increased MTT OD values (Fig. 4d), EdU staining (Fig. 4e), and colony formation (Fig. 4f) were detected in the OE-Lnc-THOR cells. These results indicate that *Lnc-THOR* overexpression promotes A172 cell growth and migration. Similarly in U251MG cells and primary human glioma cells (“Pri-1/-2”), adding *Lnc-THOR*-expressing lentivirus increased *Lnc-THOR* expression (Fig. 4g), enhancing cell survival (Fig. 4h) and proliferation (Fig. 4i).

### *Lnc-THOR* depletion activates MAGEA6-AMPK signaling in glioma cells

**Fig. 5:**
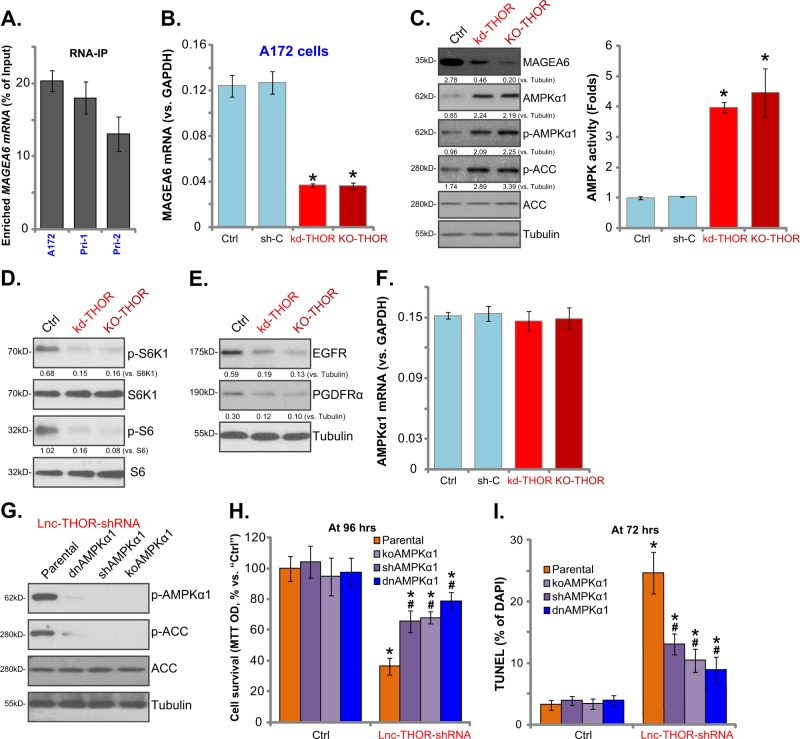
*Lnc-THOR* depletion activates MAGEA6-AMPK signaling in human glioma cells. qPCR analyses of *MAGEA6 mRNA* expression enriched by the IGF2BP1 protein in A172 cells and primary human glioma cells (**a**). The genetically modified stable A172 cells with *Lnc-THOR shRNA* (“kd-THOR,” “Seq1”), scramble non-sense control shRNA (“sh-C”), or the lenti-CRISPR/Cas9 Lnc-THOR-KO construct (“KO-THOR”) were established, and the expression of listed mRNAs (**b**, **f**) and proteins (**c**–**e**), as well as the relative AMPK activity (**c**), in the stable cells and in parental control A172 cells (“Ctrl”) were tested. Stable A172 cells with the dominant-negative AMPKα1 (“dnAMPKα1,” T172A), the AMPKα1 shRNA (shAMPKα1), the lenti-CRISPR/Cas9 AMPKα1 KO construct (“koAMPKα1”), or the parental control A172 cells (“Parental”) were further infected with/without *Lnc-THOR* shRNA virus, and the expression of listed proteins was tested by western blotting assay (**g**); Cell survival (MTT assay, **h**) and apoptosis (TUNEL staining assay, **i**) were tested. Blot data were quantified and normalized to the corresponding loading control (**c**–**e**). Data are presented as mean ± SD (*n* = 5). *p < 0.05 vs. “Ctrl” cells. ^#^*p* < 0.05 vs. “Lnc-THOR-shRNA” treatment of “Parental” cells (**h**, **i**). Experiments in this figure were repeated three times, and similar results were obtained

MAGEA3/6-TRIM28 complex is a cancer-specific ubiquitin ligase of AMPKα1^21,27,40,41^. Our previous study has shown that MAGEA6 sequesters AMPKα1 in glioma cells, causing mTORC1 overactivation and cancer cell growth. Reversely, MAGEA6 silencing inhibits human glioma cell cells via re-activation of AMPK signaling^21^. RIP assays (same experiments as Fig. 2l) confirmed the direct binding between *MAGEA6 mRNA* and the IGF2BP1 protein in A172 cells and the primary human glioma cells (Fig. 5a). Importantly, *Lnc-THOR* silencing (by “Seq1” shRNA, see Fig. 2) or KO (see Fig. 2) downregulated *MAGEA6 mRNA* (Fig. 5b) and protein (Fig. 5c) in A172 cells. Consequently, AMPKα1 protein expression and AMPK activation (AMPKα1-ACC phosphorylation) were significantly increased (Fig. 5c). AMPK activity was increased as well in *Lnc-THOR*-silenced/-KO cells (Fig. 5c).

Activated AMPK will inhibit human cancer cells via regulating its downstream effectors, causing mTORC1 inhibition^23,24,42^, autophagy induction^42–44^, and receptor tyrosine kinase (RTK) degradation^10,26^. In A172 cells, *Lnc-THOR* silencing or KO largely inhibited p70S6K1-S6 phosphorylation, indicating mTORC1 inhibition (Fig. 5d). RTKs, including EGFR and PDGFRα, were downregulated (Fig. 5e). Significantly, *AMPKα1 mRNA* levels were unchanged by *Lnc-THOR* silencing or KO (Fig. 5f). Based on these results, we propose that *Lnc-THOR* depletion activates AMPK activation possibly by downregulating AMPKα1’s ubiquitin ligase MAGEA6.

To test whether AMPK activation mediated *Lnc-THOR*-depletion-induced cytotoxicity of glioma cells, we utilized previously described genetic strategies^21^ to block AMPK activation. The lentiviral AMPKα1 shRNA, the dominant-negative AMPKα1 (“dnAMPKα1,” T172A) construct, or the lenti-CRISPR/Cas9 AMPKα1 KO construct was separately transduced to A172 cells. Stable cells were established via selection (see “Methods”). As shown, *Lnc-THOR* shRNA (“Seq1,” see Fig. 2) induced AMPK activation or AMPKα1-ACC phosphorylation was almost completely blocked by AMPKα1 shRNA, dominant-negative mutation, and KO (Fig. 5g). As a result, *Lnc-THOR* shRNA-induced glioma cell death (Fig. 5h) and apoptosis (Fig. 5i) were largely ameliorated. Therefore, AMPK activation mediates *Lnc-THOR*-depletion-induced glioma cell death.

### *Lnc-THOR* silencing is ineffective in IGF2BP1-KO glioma cells

**Fig. 6:**
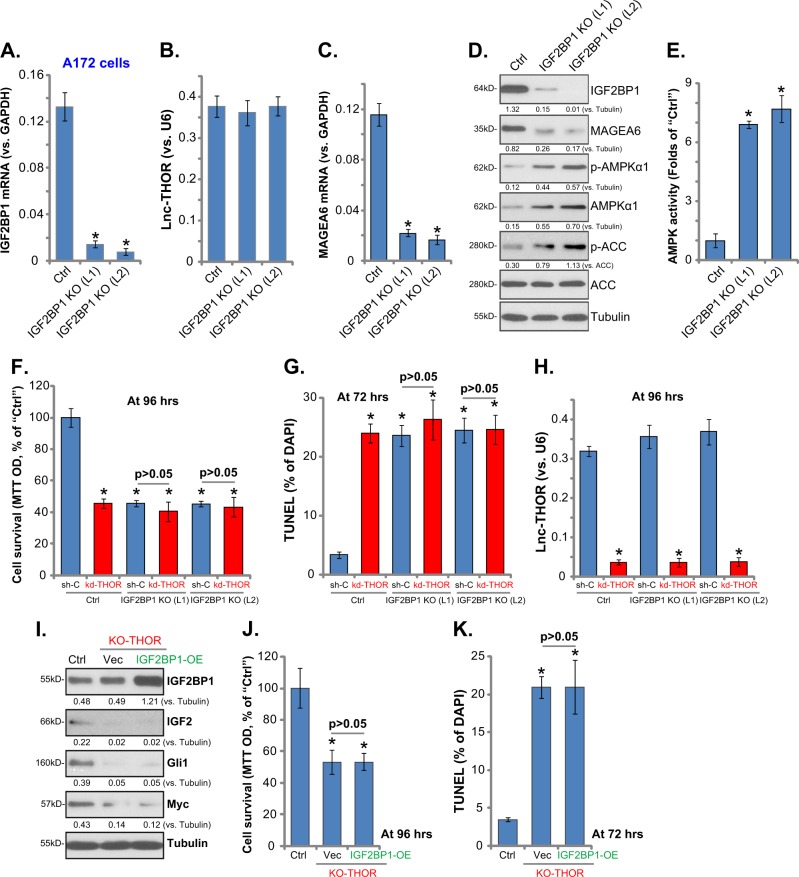
*Lnc-THOR* silencing is ineffective in IGF2BP1-knockout glioma cells. The stable A172 cells with CRISPR/Cas9-IGF2BP1-KO construct (“IGF2BP1 KO,” “L1/L2,” two lines) were established. Expression of listed mRNAs (**a**, **c**), *Lnc-THOR* (**b**), and proteins (**d**), as well as the relative AMPK activity (**e**), in IGF2BP1 KO cells and the parental control A172 cells (“Ctrl”) were tested. Cells were further infected with/without *Lnc-THOR* shRNA virus (“kd-THOR”) or the scramble control non-sense shRNA virus (“sh-C”), and cell survival (MTT assay, **f**), cell apoptosis (TUNEL assay, **g**), and *Lnc-THOR* expression (**h**, qPCR assay) were tested. Stable A172 cells with the lenti-CRISPR/Cas9 Lnc-THOR-KO construct (“KO-THOR”) were further infected with or without adenovirus encoding IGF2BP1 expression construct (IGF2BP1-OE), and stable cells were established with puromycin selection; the expression of listed proteins is shown (**i**); After culture for applied time periods, cell survival (MTT OD, **j**) and apoptosis (TUNEL staining, **k**) were tested. Blot data were quantified and normalized to the corresponding loading control (**d**, **i**). Data are presented as mean ± SD (*n* = 5). *p < 0.05 vs. “Ctrl” cells (**a**–**e**, **j**, **k**); *p < 0.05 vs. “sh-C” treatment of “Ctrl” cells (**f**–**h**). Experiments in this figure were repeated four times, and similar results were obtained

Using the CRISPR/Cas9 gene-editing method (see ref. ^36^), we established two lines of IGF2BP1 KO A172 cells (IGF2BP1 KO, “L1/L2”). qPCR results in Fig. 6a confirmed *IGF2BP1 mRNA* depletion, which did not affect the *Lnc-THOR* expression (Fig. 6b). Importantly, in IGF2BP1 KO A172 cells, *MAGEA6 mRNA* (Fig. 6c) and protein (Fig. 6d) levels were significantly downregulated, accompanied with increased AMPKα1 expression (Fig. 6d) and AMPKα1-ACC phosphorylation (Fig. 6d), as well as increased AMPK activity (Fig. 6e). Therefore, IGF2BP1 is important for MAGEA6 expression and AMPK inactivation in glioma cells.

Similar to the phenotypes of *Lnc-THOR* depletion, IGF2BP1 KO also promoted A172 cell death (Fig. 6f) and apoptosis (Fig. 6g). Importantly, adding *Lnc-THOR* shRNA lentivirus (“Seq1”) was invalid in IGF2BP1 KO cells (Fig. 6f, g), although the applied shRNA did downregulate *Lnc-THOR* in A172 cells (Fig. 6h). These results confirm that *Lnc-THOR* silencing is ineffective in IGF2BP1-KO glioma cells. Further studies demonstrated that *Lnc-THOR* KO (see Fig. 2) downregulated IGF2BP1’s targets: IGF2, Gli1, and Myc, in A172 cells, which was not affected by ectopic IGF2BP1 overexpression (Fig. 6i). Furthermore, IGF2BP1 overexpression failed to reverse *Lnc-THOR* KO-induced viability reduction (Fig. 6j) and apoptosis activation (Fig. 6k) in A172 cells.

### *Lnc-THOR* silencing or depletion inhibits A172 xenograft tumor growth in vivo

**Fig. 7:**
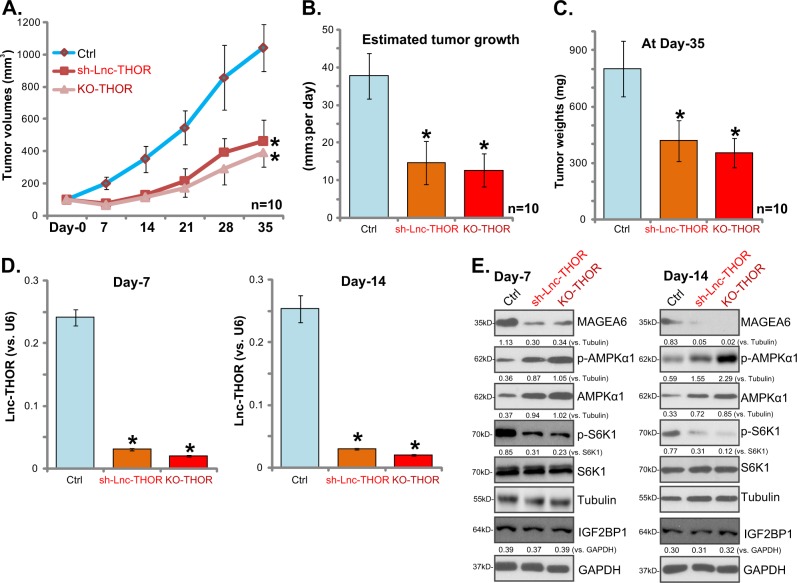
*Lnc-THOR* silencing or depletion inhibits A172 xenograft tumor growth in vivo. Parental control A172 cells (“Ctrl”), the genetically modified stable A172 cells with *Lnc-THOR* shRNA (“sh-Lnc-THOR,” “Seq1”), or the lenti-CRISPR/Cas9 Lnc-THOR-KO construct (“KO-THOR”) were injected s.c. to the flanks of female SCID mice. When the tumors reached 100 mm^3^ (10 mice per group), recordings were initiated (“Day-0”). Tumor volumes (**a**, in mm^3^) were monitored every 7 days; Daily tumor growth was calculated as described (**b**). At Day-35, tumors of all three groups were isolated and weighed individually (**c**). At Day-7 and Day-14, one tumor per group was isolated, and the expression of *Lnc-THOR* (**d**) and listed proteins (**e**) in the fresh tumor lysates was tested. Blot data were quantified and normalized to the corresponding loading control (**e**). Data are presented as mean ± SD. *p < 0.05 vs. “Ctrl” group (**a**–**c**, *n* = 10)

As described in our previous study^21^, an A172 tumor xenograft SCID mice model was established to study the potential activity of *Lnc-THOR* in vivo. The genetically modified stable A172 cells with *Lnc-THOR* shRNA (“sh-Lnc-THOR,” “Seq-1”) or lenti-CRISPR/Cas9 Lnc-THOR-KO construct (“KO-THOR”), as well as the parental control A172 cells (“Ctrl”), were inoculated via s.c. injection to the flanks of the SCID mice. Tumor volumes were recorded, and the tumor growth curve results in Fig. 7a demonstrated that A172 tumor growth was significantly inhibited with *Lnc-THOR* silencing or depletion. Estimated daily tumor growth, calculated by (estimated tumor volume at Day-35 − estimated tumor volume at Day-0)/35), was also significantly decreased in “sh-Lnc-THOR” tumors and “KO-THOR” tumors (Fig. 7b). Moreover, “sh-Lnc-THOR” tumors and “KO-THOR” tumors weighed significantly lower than “Ctrl” tumors (Fig. 7c). The body weights of the SCID mice were not significantly different between the three groups (data not shown). These results confirmed that *Lnc-THOR* silencing or depletion inhibited A172 xenograft tumor growth in vivo.

In order to test signaling changes in vivo, at Day-7 and Day-14, one tumor per group was isolated (total six tumors). Fresh tumor lysates were achieved and tested. When compared to “Ctrl” tumors, *Lnc-THOR* levels were significantly decreased in the “sh-Lnc-THOR” tumors and “KO-THOR” tumors (Fig. 7d), where MAGEA6 downregulation, AMPKα1 upregulation, and AMPK activation, as well as p-S6K1 inhibition, were detected (Fig. 7e). Total S6K1 and IGF2BP1 expression was unaffected by Lnc-THOR silencing or KO in tumor lysates (Fig. 7e). These results in vivo are therefore in line with the in vitro findings.

## Discussion

The results of the current study indicate that *Lnc-THOR* could possibly be a novel and important therapeutic target of human glioma. *Lnc-THOR* is uniquely expressed in human glioma tissues and cells. Its expression is extremely low or even undetected in normal brain tissues, as well as in normal neuronal cells/astrocytes. In established (A172 cell line) and primary human glioma cells, *Lnc-THOR* shRNA or KO potently inhibited cell survival and proliferation, while provoking cell apoptosis. Contrarily, forced overexpression of *Lnc-THOR* can further promote glioma cell growth and migration. In vivo, A172 xenograft tumors with *Lnc-THOR* silencing or KO grew significantly slower than control tumors in SICD mice. These results are in line with recent findings proposing *Lnc-THOR* as a novel therapeutic oncotarget for many human cancers^16–20,45^.

MAGE-TRIM28 complex is a cancer-specific AMPKα1 ubiquitin ligase^21,27,40,41^. We have previously shown that MAGEA6, one of the key AMPKα1’s ubiquitin ligase^21,27,40,41^, is uniquely expressed in human glioma tissues and cells, responsible for AMPKα1 degradation and AMPK inhibition. Contrarily, MAGEA6 silencing/depletion restored AMPKα1 expression and induced AMPK activation, causing downstream mTORC1 inactivation and glioma cell death^21^. The regulatory mechanism of MAGEA6 expression in glioma is elusive. The results of this study suggest that *Lnc-THOR*-IGF2BP1 association is important for MAGEA6 expression in glioma cells. The RIP results show that *MAGEA6 mRNA* directly binds to the IGF2BP1 protein in A172 cells and the primary glioma cells. Significantly, *Lnc-THOR* silencing/KO or IGF2BP1 KO induced MAGEA6 degradation (both mRNA and protein), AMPKα1 protein accumulation, and AMPK activation in A172 glioma cells. These results suggest that *Lnc-THOR*-IGF2BP1 complex is important for MAGEA6 expression, causing AMPKα1 degradation and AMPK inactivation in human glioma cells.

**Fig. 8:**
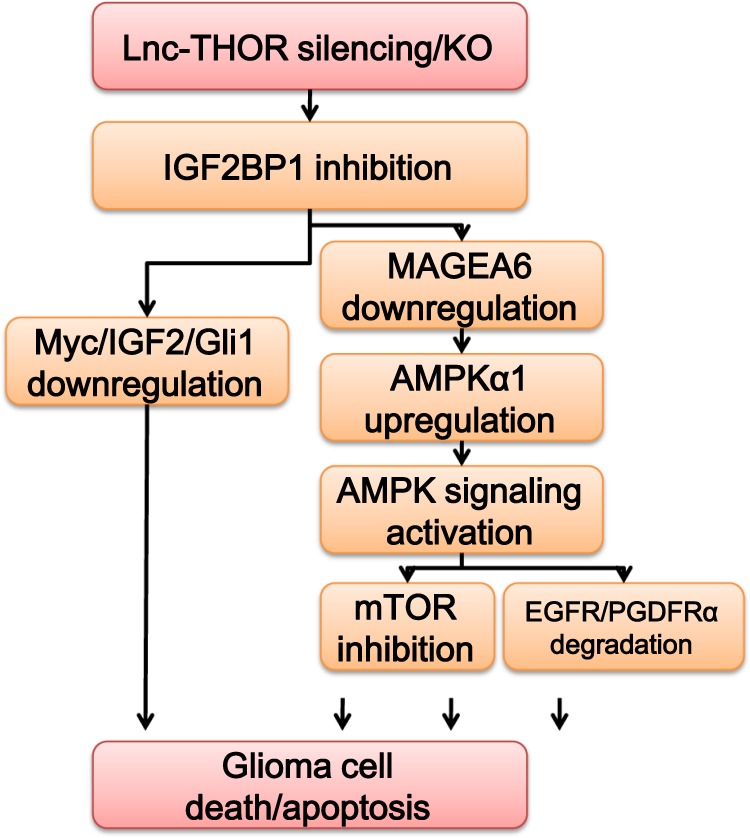
The proposed signaling pathway of this study

Our study^21^ and others have implied that forced activation of AMPK signaling can induce human cancer cell apoptosis via regulating its downstream effectors, including mTORC1 inhibition^23,24,42^, autophagy induction^42–44^, and RTK (EGFR, PDGFR, etc) degradation^10,26^. We provided evidences here to support that AMPK activation mediates, at least in part, *Lnc-THOR*-depletion-induced glioma cell death. In A172 cells, *Lnc-THOR* silencing/KO induced MAGEA6 degradation, AMPKα1 elevation, and AMPK signaling activation, causing mTORC1 inhibition and EGFR–PDGFR degradation and eventually cell apoptosis. Similarly, IGF2BP1 KO also activated MAGEA6-AMPK signaling in A172 cells. Importantly, AMPK inactivation, by AMPKα1 shRNA, KO, or dominant-negative mutation, attenuated *Lnc-THOR* shRNA-induced A172 cell apoptosis. Significantly, AMPK blockage failed to completely reverse *Lnc-THOR* shRNA-induced cytotoxicity in A172 glioma cells, suggesting that both AMPK-dependent and AMPK-independent mechanisms are responsible for *Lnc-THOR*-silencing-induced glioma cell death (see Fig. 8, the proposed signaling pathway of the study). Therefore, although further studies are needed to explore the detailed underlying mechanisms, here we propose that *Lnc-THOR*-IGF2BP1 association is vital for MAGEA6 expression and AMPK inactivation in human glioma cells (Fig. 8).

## Conclusion

*Lnc-THOR* depletion activates MAGEA6-AMPK signaling and inhibits human glioma cell survival.
